# Negative workplace gossip and turnover intention among Chinese rural preschool teachers: The mediation of ego depletion and the moderation of *bianzhi*

**DOI:** 10.3389/fpsyg.2022.1034203

**Published:** 2022-12-02

**Authors:** Can He, Hua Wei

**Affiliations:** ^1^College of Education Science, Hubei Normal University, Huangshi, China; ^2^Normal College, Qingdao University, Qingdao, China

**Keywords:** negative workplace gossip, ego depletion, turnover intention, *bianzhi*, rural preschool teachers

## Abstract

**Introduction:**

In China, the high turnover rates of teachers have become one of the biggest obstacles to the development of rural preschool education. Objective: Based on the social information processing theory and the strength model of selfcontrol, this study examined the relationship between negative workplace gossip and turnover intention and the role of ego depletion and bianzhi in this relationship.

**Methods:**

The questionnaire method was applied, and 411 rural preschool teachers in Hubei Province, China, participated in the survey.

**Results:**

The correlation results showed that negative workplace gossip was positively correlated with ego depletion, and ego depletion was positively correlated with turnover intention. After controlling for age, negative workplace gossip positively predicted turnover intention. The mediation analysis indicated that ego depletion played a mediating role in the relationship between negative workplace gossip and turnover intention. Moreover, the mediation effect was moderated by bianzhi. Negative workplace gossip had a stronger effect on the ego depletion of teachers without bianzhi than on that of teachers with bianzhi.

**Conclusion:**

The current research is the first to clarify that ego depletion mediates the relationship between negative workplace gossip and turnover intention, and the mediation effect is moderated via bianzhi. These findings expand our understanding of the influential factors and formation mechanisms of turnover intention. In practice, this study provides a novel perspective for policymakers and administrators to reduce the turnover intention among rural preschool teachers in China.

## Introduction

The high turnover rates of preschool teachers impair children’s early development, threaten the stability of teaching staff, and even hinder the improvement of preschool education (Wells, 2015; Schaack et al., 2022). However, high turnover rates of preschool teachers are increasingly common around the world (Hong, 2010; Wells, 2015; Schaack et al., 2022). In the United States, approximately 15–30% of early childhood teachers quit their jobs every year (Whitebook et al., 2014), while the annual turnover rate of some childcare institutions in Australia is as high as 60% (Jovanovic, 2013). In China, preschool institutions are also facing the same problem. A survey of some preschools in Beijing found that one-third of teachers left their jobs annually (Feng et al., 2017), and the situation is even worse in rural areas (Li and Gong, 2020). The poor stability and severe shortage of preschool teachers have long been a problem in rural China. According to the preschool staff standards promulgated by the Ministry of Education of the People’s Republic of China, the minimum ratio of full-time preschool staff to children should be 1:7. However, a survey in 12 Chinese provinces showed that only 16.9% of rural preschools met the standard (Yu et al., 2017), and the cumulative shortage of rural preschool teachers reached 640,000 (Liu and Bai, 2021). The high turnover rate further exacerbates teacher shortage and challenges the building of the rural preschool teacher workforce in China. To alleviate preschool teacher attrition, it is of great significance to understand the contributing factors of turnover intention.

Turnover intention, which refers to the inclination to quit one’s job (Tett and Meyer, 1993), has been shown to be the strongest predictor of actual turnover behavior (Griffeth et al., 2000). Previous studies have shown that various individual and environmental factors affect the turnover intention of rural preschool teachers (Tang et al., 2015; Ye et al., 2018; Wang et al., 2022). As environmental factors, deviant workplace behaviors have been extensively explored in organizational behavior research in recent years and have shown to be important antecedents of employee turnover intention. Although the effects of interpersonal deviance (such as workplace bullying and workplace incivility) on the targets’ turnover intention have been analyzed (Mahfooz et al., 2017; Malik et al., 2018; Favaro et al., 2021; Moon and Morais, 2022), to our knowledge, no studies have yet examined the effects of negative workplace gossip.

Negative workplace gossip is defined as the informal, negative and evaluative discussion or the spreading of rumors about an absent third member of the organization (Chandra and Robinson, 2009; Cheng et al., 2020). Compared to other interpersonal deviance (such as workplace bullying and workplace incivility), negative workplace gossip is significantly different. It is covert and indirect in nature, occurring in the absence of the target (Foster, 2004; Wu et al., 2018). In this sense, negative workplace gossip is different from other forms of interpersonal maltreatment which typically include both overt and covert behaviors (Duffy et al., 2002). Thus, negative workplace gossip can be regarded as a form of indirect aggression (Beersma and Van Kleef, 2012; Wu et al., 2018; Ullah et al., 2021). Unlike other forms of direct interpersonal aggression, it is difficult for the target to trace the source or verify the content of the gossip, making it susceptible to more ambiguity and confusion (Wu et al., 2018; Ullah et al., 2021). Furthermore, negative workplace gossip is ubiquitous in all organizations where individuals often intentionally overhear, disseminate, or participate in gossip about other colleagues in conversation (Kong, 2018; Liu et al., 2020; Ullah et al., 2021). Given the above-discussed characteristics, negative workplace gossip may trigger more detrimental consequences. Previous studies have shown that negative workplace gossip can cause great distress to the targets and have a negative impact on their work behaviors and attitudes (Ye et al., 2019; Cheng et al., 2020; Yao et al., 2020; Ullah et al., 2021; Zong et al., 2021).

Despite these findings, to our knowledge, no studies have been conducted on the relationship between negative workplace gossip and turnover intention. Negative gossip is pervasive in organizations. Furthermore, China is a collectivist country where interpersonal relationships occupy a pivotal position. Whether the turnover intention of rural preschool teachers is affected by negative workplace gossip is a topic worthy of in-depth discussion. According to the social information processing theory (Salancik and Pfeffer, 1978), the negative social atmosphere caused by negative workplace gossip affects the perceptions, attitudes, and behaviors of the targets, leading to their sense of exclusion (Kuo et al., 2018) and reducing their organizational identification (Ye et al., 2019), which may trigger their intention to leave. Therefore, the present study attempts to explore the impact of negative workplace gossip on turnover intention among rural preschool teachers in Chinese cultural context.

One possible way negative workplace gossip affects turnover intention is by influencing ego depletion. Previous studies have mainly explored the influencing mechanisms of deviant workplace behaviors on turnover intention from the perspective of cognition and stress (Mahfooz et al., 2017; Salin and Notelaers, 2017; Malik et al., 2018; Bentley et al., 2021; Moon and Morais, 2022; Schwepker Jr and Dimitriou, 2022). However, the functioning mechanism has not been analyzed from an integrated perspective. In fact, both negative cognitive processing and stress regulation consume limited self-control resources, which will result in ego depletion (Baumeister et al., 2007). According to the strength model of self-control (Muraven and Baumeister, 2000), the low-quality interpersonal treatment behaviors such as verbal attacks and personal insults contained in negative workplace gossip may make the gossip targets consume limited self-control resources to cope with negative emotions, cognitive dissonance, and psychological pressure, resulting in their ego depletion (Cheng et al., 2020; Ullah et al., 2021). Ego depletion may further reduce the targets’ self-regulation ability and undermine their ability to control the external environment, triggering work alienation (Cui et al., 2022) and reducing organizational commitment (Mulki et al., 2008; Dong et al., 2022), which will lead to their turnover intention. Furthermore, previous study has verified that ego depletion mediates the relationship of negative workplace gossip and employee proactive behaviors (Ullah et al., 2021). The strength model of self-control provides an integrated framework to explain individuals’ self-regulation and behavioral choices in response to corporate events from the perspective of self-control resources. In light of this framework, this study explores the mediating effect of ego depletion in the relationship between negative workplace gossip and turnover intention.

In addition, although negative workplace gossip can trigger ego depletion, the effect may be moderated by many variables. Previous studies have mainly explored the boundary conditions pertaining to the influence of negative workplace gossip on ego depletion in terms of individual factors such as emotional intelligence and sensitivity to interpersonal mistreatment (Cheng et al., 2020; Ullah et al., 2021) but have ignored the role of contextual factors. However, the impact of negative workplace gossip on individuals’ ego depletion is inevitably related to contextual factors. It has been shown that social identity can weaken the ego depletion effect (Loschelder and Friese, 2016). It is likely that *bianzhi*, an important contextual factor affecting the social identity of Chinese teachers, also acts as a buffer. In China, *bianzhi* is regarded by teachers as the guarantee of a lifelong job, which is nearly equivalent to tenure in the American education system (Hu et al., 2014, 2016). To some extent, *bianzhi* has become an important status symbol, which can increase the organizational identification of preschool teachers (Liu et al., 2021). Teachers with *bianzhi* have higher levels of organizational identification and thus are less likely to experience ego depletion when facing negative workplace gossip. Wang et al. (2022) found that *bianzhi* could moderate the negative effect of work pressure on the turnover intention of rural preschool teachers. However, no studies have examined whether *bianzhi* buffers the consequences of negative workplace gossip. Therefore, *bianzhi* was introduced into the present study to investigate its moderating effect on the relationship of negative workplace gossip with ego depletion and turnover intention. In summary, based on the social information processing theory and the strength model of self-control, the present study takes ego depletion as the mediator and *bianzhi* as the moderator, and comprehensively discusses the functioning mechanisms of negative workplace gossip on turnover intention and the boundary conditions so as to provide theoretical guidance and managerial implications for addressing rural preschool teacher attrition in China.

### Relationship between negative workplace gossip and turnover intention

As an old Chinese saying goes, “gossip is a fearful thing, and public clamor can melt metal.” As a highly salient workplace stressor, negative workplace gossip can exert a series of negative effects on gossip targets. Previous studies have shown that negative workplace gossip can cause varying degrees of damage to employees’ dignity and reputation, which further affects their attitudes and behaviors in the workplace. For example, negative workplace gossip can cause emotional exhaustion (Wu et al., 2018), reducing proactive behaviors (Wu et al., 2018) and service performance (Ye et al., 2019), inhibiting organizational citizenship behaviors (Wu et al., 2018), and increasing workplace ostracism (Kuo et al., 2018) and political acts (Cheng et al., 2020). The social information processing theory proposes that social information from the surrounding environment influences individuals’ attitudes and behaviors (Salancik and Pfeffer, 1978). Negative workplace gossip creates a negative social atmosphere and public opinion environment for the targets, and this interpersonal environment affects the targets’ perceptions, attitudes and behaviors. Individuals who experience negative workplace gossip feel that they are being excluded by other members of the organization (Kuo et al., 2018), which will lower their perceived insider status (Kong, 2018), and reduce their organizational identification (Ye et al., 2019; Yao et al., 2020), ultimately leading to turnover intention. For preschool teachers, the public often has high professional ethics requirements. In addition, most rural preschools in China are small in scale, and faculty members will frequently meet in their daily work. Therefore, negative workplace gossip in rural preschools may be more destructive. Relevant studies on organization behaviors have also confirmed that deviant workplace behaviors similar to negative workplace gossip, such as workplace bullying, workplace ostracism, and workplace incivility, can lead to turnover intention (Mahfooz et al., 2017; Malik et al., 2018; Favaro et al., 2021; Moon and Morais, 2022). Therefore, we propose H1: Negative workplace gossip is positively associated with turnover intention among rural preschool teachers.

### Mediation effect of ego depletion

The strength model of self-control points out that self-control strength is necessary for the executive component of the self to function, and all self-control operations draw on the same resources (Muraven and Baumeister, 2000). The resources are limited and vulnerable to becoming depleted. When demands on self-control resources are frequently made over time, they can become depleted. Baumeister et al. termed the state of resource reduction as ego depletion (Stucke and Baumeister, 2006; Baumeister et al., 2007), which refers to self-control resource diminishment following effortful acts of will (Hagger et al., 2010). According to the strength model of self-control and previous research, ego depletion may be a mediating variable for the relationship between negative workplace gossip and turnover intention. Negative workplace gossip may consume individuals’ psychological resources and lead to ego depletion. The mechanisms are as follows. First, a negative organizational atmosphere full of gossip or rumors can increase employees’ job insecurity (Bordia et al., 2006). If job stability and continuity are threatened, individuals will deploy many cognitive resources to cope with work-related uncertainty (Muraven and Baumeister, 2000). Moreover, the targets may bother themselves by thinking about whether the gossip was attributed to their inappropriate behaviors. This thinking process may last for a long time and consume lots of cognitive resources, leading to ego depletion (Cheng et al., 2020; Ullah et al., 2021). Second, negative workplace gossip can trigger the targets’ negative emotions (Wu et al., 2018; Babalola et al., 2019). They may feel embarrassed by the disclosure of private information and despair over the damage to their reputation. In addition, they may have difficulty determining who is to blame, and they may have to suppress negative emotions. Regulating these negative emotions further consumes their limited psychological resources, thereby accelerating ego depletion.

Ego depletion may lead to turnover intention. According to the strength model of self-control, meeting work requirements and accomplishing work goals will consume self-control resources. Ego depletion may reduce self-regulation ability and lead to cognitive biases, which make individuals underestimate their ability to control the external environment, thus triggering work alienation (Cui et al., 2022). Individuals with higher levels of work alienation isolate themselves from the work environment and reduce their social interactions at work, and then their desire to work hard for the organization and maintain stable relationships with the organization members decreases, leading to a decline in organizational commitment (Mulki et al., 2008; Dong et al., 2022). According to the casual models of employee turnover (Price, 1977; Steers and Mowday, 1981), organizational commitment is an important predictor of employee turnover, and lower organizational commitment indicates a higher turnover intention. Ego depletion causes psychological detachment of individuals from work and social isolation from organization members, which decreases their organizational commitment and ultimately leads to turnover intention. Previous studies have also shown that ego depletion causes a series of negative consequences, such as absenteeism (Diestel and Schmidt, 2011). Accordingly, we propose the H2: Negative workplace gossip may relate to turnover intention through the mediating role of ego depletion. Negative workplace gossip is positively correlated with ego depletion, and ego depletion is positively correlated with turnover intention.

### Moderation effect of *bianzhi*

The ego depletion effect can be moderated by many variables, and *bianzhi* may be one of the moderators. *Bianzhi* refers to an officially established post of teachers assigned by the local department of education, which is nearly equivalent to tenure in the American education system (Hu et al., 2014, 2016). In China, *bianzhi* is a symbol of teachers’ identity, making it extremely important for teachers. *Bianzhi* is accompanied by a series of benefits, such as lifelong employment, stable salaries, public housing subsidies, and reliable medical insurance. Although there may be no difference in the work content between teachers with *bianzhi* and those without *bianzhi*, their occupational stability, salaries, subsidies and social status are quite different (Liu et al., 2021). Accordingly, rural preschool teachers in China can be divided into two categories: teachers with and without *bianzhi*. Due to the large *bianzhi* gap among rural preschool teachers, the number of teachers without *bianzhi* accounts for the vast majority of rural preschool teachers. Based on relevant theories and a literature review, this study suggests that *bianzhi* may moderate the first half of the mediating pathway of “negative workplace gossip → ego depletion → turnover intention.” When confronted with negative workplace gossip, preschool teachers with and without *bianzhi* may have different cognitive responses. The social identity theory states that people tend to identify with a group or organization to elevate their self-esteem (Tajfel et al., 1971). When people acquire group identities, they unconsciously identify with the group or organization to which they belong and tend to give more positive evaluations of group or organization members (Tajfel and Turner, 1982). Relevant research confirms that individuals tend to forgive in-group members (Yao et al., 2014). For preschool teachers with *bianzhi*, *bianzhi* serves as an institutional identity guarantee that increases their sense of organizational identification (Liu et al., 2021). According to the social identity theory, preschool teachers with *bianzhi* may treat negative gossip from organization members with more tolerance, thus weakening the impact of negative workplace gossip on ego depletion. In contrast, teachers without *bianzhi* may be more sensitive to negative workplace gossip and thus have to deploy more cognitive resources for self-regulation, which ultimately leads to ego depletion. Moreover, social identity can counteract the ego depletion effect (Loschelder and Friese, 2016).

The emotional resources that teachers with and without *bianzhi* use to cope with ego depletion may be different. Previous studies have shown that positive emotions can counteract the ego depletion effect because they can restore the body to a neutral state, eliminate the negative physiological effects of negative emotions and thus replenish self-control resources (Tice et al., 2007). The positive emotional experience of preschool teachers with *bianzhi* is significantly greater than that of teachers without *bianzhi* (Wei, 2014), so they have more emotional resources to mitigate the impact of negative workplace gossip on ego depletion. Negative workplace gossip may have a smaller effect on the ego depletion of teachers with *bianzhi* than those without *bianzhi*. Therefore, we propose H3: *bianzhi* moderates the relationship between negative workplace gossip and ego depletion. Negative workplace gossip has a weaker effect on the ego depletion of teachers with *bianzhi* than on that of teachers without *bianzhi*.

### The present study

**Figure 1:**
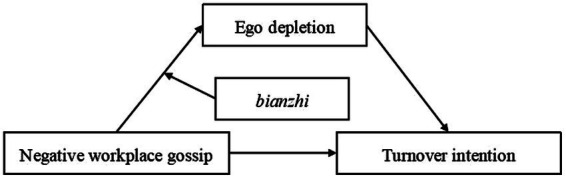
The proposed moderated mediation model.

The present study aims to establish a moderated mediation model to examine the impact of negative workplace gossip on turnover intention and test the mediating effect of ego depletion and the moderating effect of *bianzhi* (Figure 1).

## Materials and methods

### Participants and procedure

The present study was approved by the Ethics Committee for Scientific Research of Hubei Normal University. Convenience sampling was adopted to conduct this survey. Due to the need to prevent and control the epidemic, data were collected through Questionnaire Star, an online questionnaire platform. Specifically, this survey was organized by the Huangshi Municipal Bureau of Education, and the questionnaires were distributed to preschool teachers through the principals. All participants were asked to complete the questionnaires truthfully and independently in an anonymous way. In addition, informed consent was obtained from the teachers before the test. We recruited 450 rural preschool teachers from various rural preschools in Hubei Province during the fall semester of 2021. After eliminating questionnaires completed too quickly (<3 min) and those with incomplete answers, we obtained 411 valid questionnaires. All participants were female and full-time teachers. Among the participants, 50 (12.2%) had *bianzhi,* and 361 (87.8%) did not have *bianzhi* (median age = 32.94, SD = 7.11).

### Measures

#### Negative workplace gossip

Negative workplace gossip was evaluated with the Chinese version of a three-item scale developed by Chandra and Robinson (2009). The Chinese version has been proven to have good validity (Xing et al., 2021). A sample item was “As recently as 1 month ago, false allegations were made about you.” Participants answered questions based on a five-point scale from 1 (never) to 5 (daily), with higher scores suggesting higher exposure to negative workplace gossip. In this study, Cronbach’s α for the scale was 0.94.

#### Ego depletion

Ego depletion was measured with five items from a scale developed by Twenge et al. (2004), which has been used in the Chinese context (Cheng et al., 2020). Participants needed to determine the extent to which each statement represented how they felt at that moment (e.g., I feel drained) using a 4-point Likert scale (from 1 = not at all to 4 = very much). Higher scores indicate higher levels of ego depletion. In the present study, Cronbach’s α for the scale was 0.86.

#### Turnover intention

Turnover intention was measured with a three-item scale developed by Konovsky and Cropanzano (1991). A sample item was “How likely is it that you will look for a job outside this organization during the next year?,” and responses were rated from “1 = very unlikely” to “5 = very likely.” Higher scores indicate stronger willingness of individuals to quit the current job. The scale has good reliability and validity in Chinese samples (Shu and Ye, 2019). In the present study, Cronbach’s α for the scale was 0.91.

### Data analyzes

All data were inputted and analyzed by SPSS 25.0. First, descriptive statistics and correlation analysis were performed. Second, the moderated mediation was conducted with the SPSS Macro PROCESS developed by Hayes (2018). Model 4 in the PROCESS template was used to test the mediation effect, and Model 7 in the template was used to test the moderated mediation effect. In the present study, all continuous variables were standardized before regression analyzes. In addition, *bianzhi* was translated into a dummy variable. Teachers without *bianzhi* were coded as 0, while teachers with *bianzhi* were coded as 1. When conducting the PROCESS Macro, we generated 95% bias-corrected accelerated confidence interval (CI) based on 5,000 bootstrap samples to calculate the indirect effect, and the 95% CI without zero indicated statistical significance. Additionally, age was the control variable in all regression models.

## Results

### Common method deviation test

In this study, the structural equation model was used to conduct the confirmatory factor analysis to test common method variance (Harris and Mossholder, 1996). We tested a one-factor model by Mplus 8.3, and the fit indices suggested that the model did not fit the data well (*X*^2^/df = 35.99, RMSEA =0.29, CFI = 0. 52, and TLI = 0. 39). The results indicated that common method variance did not influence this study.

### Preliminary analyzes

The means, standard deviations, and Pearson correlation coefficients for all variables are presented in Table 1. As Table 1 shows, negative workplace gossip was positively associated with turnover intention (*r* = 0.32, *p* < 0.01) and ego depletion (*r* = 0.25, *p* < 0.01); ego depletion was positively associated with turnover intention (*r* = 0.55, *p* < 0.01).

**Table 1:** Means, standard deviations and correlations for the main variables.

Variable	*M*	SD	1	2	3	4
1. NWG	1.47	0.76	−			
2. Ego depletion	1.90	0.56	0.25^**^	−		
3. *Bianzhi*	0.12	0.33	0.09	−0.07	−	
4. Turnover intention	2.45	0.85	0.32^**^	0.55^**^	−0.16^**^	−

*N* = 411. *Bianzhi* was dummy coded such that 0 = teachers without *bianzhi* and 1 = teachers with bianzhi. NWG, negative workplace gossip. ^**^p < 0.01.

### Testing for the mediating role of ego depletion

Model 4 of the PROCESS macro (Hayes, 2018) was chosen to examine the possible association between negative workplace gossip and turnover intention as well as the potential mediating effect of ego depletion. As illustrated in Table 2, negative workplace gossip positively predicted turnover intention (*β* = 0.33, *p* < 0.01); negative workplace gossip positively predicted ego depletion (*β* = 0.24, *p* < 0.01); ego depletion positively predicted turnover intention (*β* = 0.50, *p* < 0.01). The bias-corrected bootstrapping mediation test indicated that the process by which negative workplace gossip predicted turnover intention through ego depletion was significant, with indirect effect = 0.12, boot *SE* = 0.04, 95% CI = [0.05, 0.19]. Ego depletion partially mediated the association between negative workplace gossip and turnover intention. In addition, the mediation effect accounted for 36.36% of the total effect. The results of the mediation analysis support H1 and H2.

**Table 2:** Mediation analyzes.

	Equation 1 (Turnover intention)			Equation 2 (Ego depletion)			Equation 3 (Turnover intention)		
	*β*	SE	*t*	*β*	SE	*t*	*β*	SE	*t*
Age	0.01	0.01	0.95	−0.01	0.01	−0.79	0.01	0.01	1.58
NWG	0.33	0.05	7.00^**^	0.24	0.05	4.97^**^	0.21	0.04	5.02^**^
Ego depletion							0.50	0.04	12.12^**^
*R^2^*	0.11			0.06			0.34		
*F*	24.49^**^			13.49^**^			71.14^**^		

NWG, negative workplace gossip. ^**^p < 0.01.

### Testing for moderated mediation

**Figure 2:**
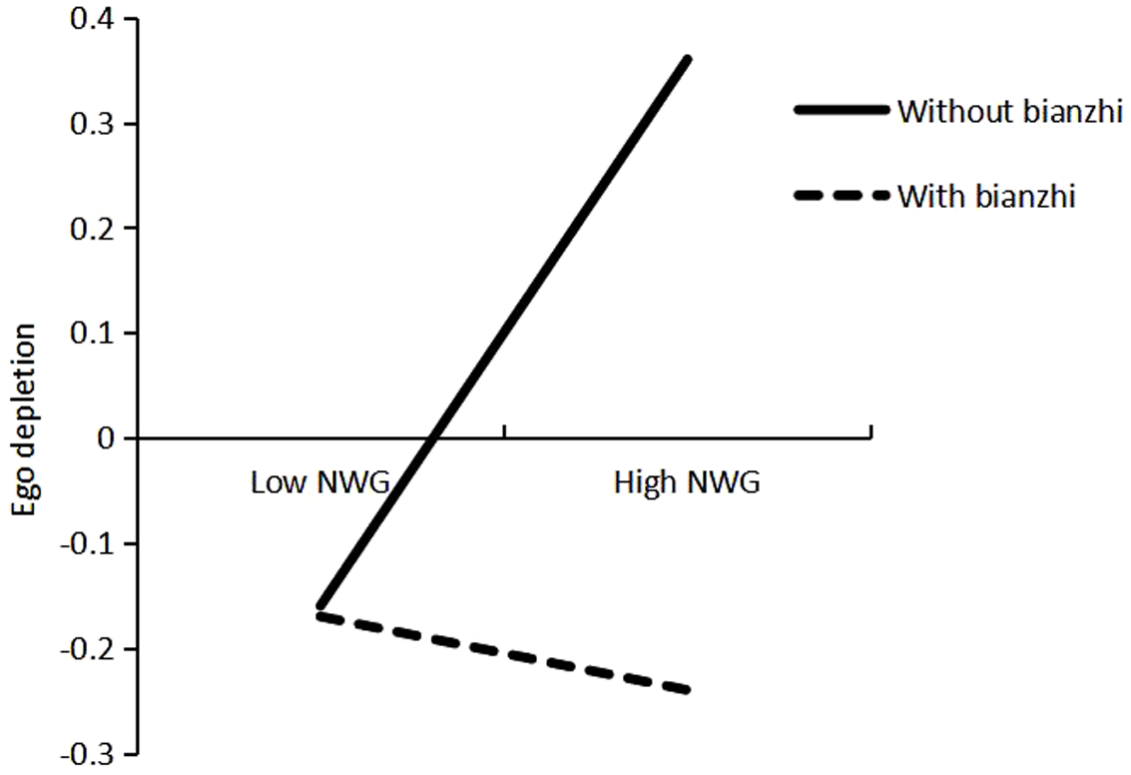
Interaction between negative workplace gossip and *bianzhi* on ego depletion.

Model 7 of the PROCESS macro (Hayes, 2018) was constructed to investigate whether *bianzhi* moderated the association between negative workplace gossip and ego depletion. The results of the moderation analysis are presented in Table 3. The regression model indicated that the interaction between negative workplace gossip and *bianzhi* was associated with ego depletion (*β* = −0.36, *p* < 0.01). Simple slope tests revealed that for preschool teachers with *bianzhi*, the relationship between negative workplace gossip and ego depletion was not significant (*B*simple = −0.04, *t* = −0.37, *p* > 0.05, CI = [−0.25, 0.17]), while for preschool teachers without *bianzhi*, the relationship between negative workplace gossip and ego depletion was significant (*B*simple = 0.32, *t* = 5.97, *p* < 0.01, CI = [0.21, 0.42]). Figure 2 illustrates the interaction plot. These analyzes indicate that *bianzhi* moderates the indirect effect of negative workplace gossip on the turnover intention of preschool teachers without *bianzhi* but not that of preschool teachers without *bianzhi*. Therefore, H3 was supported.

**Table 3:** Moderated mediation analyzes.

	Equation 1 (Ego depletion)			Equation 2 (Turnover intention)		
	*β*	SE	*t*	*β*	SE	*t*
Age	−0.01	0.01	−1.08	0.01	0.01	1.58
NWG	0.32	0.05	5.79^**^	0.21	0.04	5.02^**^
*Bianzhi*	−0.24	0.15	−1.62			
NWG × *Bianzhi*	−0.36	0.12	−2.97^**^			
Ego depletion				0.50	0.04	12.12^**^
*R^2^*	0.09			0.34		
*F*	10.25^**^			71.14^**^		

NWG, negative workplace gossip. ^**^p < 0.01.

## Discussion

Drawing on the social information processing theory and the strength model of self-control, we explored the relationship between negative workplace gossip and turnover intention among rural preschool teachers in China and examined the mediating role of ego depletion and the moderation effect of *bianzhi*. We found that negative workplace gossip was positively associated with rural preschool teachers’ turnover intention, indicating that negative workplace gossip was an important risk factor for turnover intention. When encountering negative workplace gossip, rural preschool teachers may feel that they are “out-group members” excluded by the organization (Ellwardt et al., 2012), and their organizational identification may decrease accordingly. In addition, they are inclined to keep silent to avoid confrontation (Brady et al., 2017), leading to the accumulation of dissatisfaction, which further increases turnover intentions. It is also worth noting that negative workplace gossip affected turnover intention through the mediation effect of ego depletion, that is, negative workplace gossip led to the formation of turnover intention by increasing ego depletion. Negative workplace gossip may cause the gossip targets to deploy limited psychological resources to cope with negative emotions, cognitive dissonance, and psychological pressure, resulting in ego depletion (Cheng et al., 2020; Ullah et al., 2021). Once the targets’ resources are depleted, their self-regulation ability may decrease, and they will underestimate their abilities to control the external environment, which will cause work alienation and lower organizational commitment (Cui et al., 2022; Dong et al., 2022), ultimately leading to turnover intention. Furthermore, we found that *bianzhi* moderated the relationship between negative workplace gossip and ego depletion. The indirect effect was stronger for rural preschool teachers without *bianzhi* than for those with *bianzhi*. With institutional identity affirmation and security, teachers with *bianzhi* tend to have higher organizational identification. According to the social identity theory, teachers with *bianzhi* may be more tolerant toward negative gossip from members of their organizations, thereby weakening the effect of negative workplace gossip on ego depletion.

### Theoretical contributions

This study provides several theoretical implications. First, to our knowledge, this study may be the first attempt to explore the effect of negative workplace gossip on the gossip targets’ turnover intention among rural preschool teachers. It verifies the negative impact of negative workplace gossip on employees’ work attitudes (Dlamani et al., 2018; Ye et al., 2019; Yao et al., 2020) and expands the research scope of negative workplace gossip. Previous studies have shown that interpersonal deviance, such as workplace bullying and workplace incivility, can increase employees’ turnover intention (Mahfooz et al., 2017; Malik et al., 2018; Favaro et al., 2021; Moon and Morais, 2022). Negative workplace gossip is more common than interpersonal deviance explored in previous studies and is characterized by subjectivity, concealment, rapid spread, and difficulty in source tracing (Foster, 2004; Wert and Salovey, 2004), and therefore extensive attention should be given to its negative effect on employees and organizations. China is a collectivist country that emphasizes interpersonal relationships, which play a pivotal role in people’s lives. These results suggest that we should pay attention to the negative effects of negative workplace gossip and put forward coping strategies to reduce the high turnover rates of rural preschool teachers from the perspective of interpersonal relationships.

Second, this study explains the internal influencing mechanism of negative workplace gossip on turnover intention and further verifies the mediating role of ego depletion in the consequences of negative workplace gossip (Cheng et al., 2020; Ullah et al., 2021; Xing et al., 2021). Previous studies mainly explain the impact of deviant workplace behaviors on turnover intention from the perspectives of cognition (Salin and Notelaers, 2017; Malik et al., 2018; Moon and Morais, 2022) and stress (Mahfooz et al., 2017; Bentley et al., 2021; Schwepker and Dimitriou, 2022) based on the social exchange theory and the conservation of resources theory. The present study, based on the strength model of self-control, reveals the relationship between negative workplace gossip and turnover intention from the perspective of ego depletion for the first time, which deepens the understanding of the psychological mechanisms of the consequences of negative workplace gossip. In addition, although negative cognition and stress themselves can lead to turnover intention (Lan et al., 2019; Zhang et al., 2019), negative cognitive processing and stress regulation also consume limited self-control resources (Baumeister and Vohs, 2007) and further increase the tendency to quit. Therefore, taking ego depletion as a mediator provides a new integrated perspective for the study on the mechanism by which negative workplace gossip affects turnover intention. In the future, mediators such as cognition and stress examined in previous studies can be incorporated into the framework of ego depletion. From the perspective of practical intervention, coping with ego depletion is more feasible than changing cognition and releasing stress since ego depletion can be effectively relieved in a short period of time by rest, sleep, etc., (Muraven and Baumeister, 2000). Taking ego depletion as the mediating variable is of more practical significance to intervene in the effect of workplace negative gossip on turnover intention. In conclusion, this study applies the strength model of self-control to investigate the consequences of negative workplace gossip and the formation mechanism of turnover intention. It not only first reveals the relationship between negative workplace gossip and turnover intention from the perspective of ego depletion but also promotes research on the mechanism of the consequences of negative workplace gossip and expands the application scope of the strength model of self-control to a certain extent. This study provides a theoretical direction for research on the casual models of employee turnover.

Third, this study reveals for the first time that *bianzhi*, a particular work resource, can also buffer the effect of negative workplace gossip on ego depletion, which further verifies that work resources can weaken the consequences of negative workplace gossip. Previous studies have shown that the effects of negative workplace gossip vary depending on work resources (Gao, 2018; Xie et al., 2019). According to the conservation of resources theory (Hobfoll, 1989; Halbesleben et al., 2014), inclusive leadership, organizational support, and *bianzhi* are valuable work resources that can help individuals effectively cope with stressful events at workplaces. Different from subjectively perceived work resources such as inclusive leadership and organizational support, *bianzhi* is a relatively objective work resource. The intervention of *bianzhi* is more feasible from the perspective of government macro-management. The department of education can directly intervene in the supply of *bianzhi* for rural preschool teachers, while direct intervention in leadership styles and organizational support is difficult. Therefore, taking *bianzhi* as the moderator helps us deal with rural preschool teacher attrition from a more macroscopic perspective. Based on the results of previous studies and the present study, it can be concluded that both subjective and objective resources have similar buffering effects. Therefore, future research can further examine the effects of objective job resources such as salary and performance. In addition, this study enriches research on the moderating factors of the ego depletion effect. Previous studies have mainly explored the boundary conditions of ego depletion from the perspective of subjective factors such as personality traits, emotions, and cognition (Hagger et al., 2010). To our knowledge, the present study reveals for the first time the protective effect of *bianzhi* on self-control resources from the perspective of objective factors, which lays the foundation for future research. It is worth noting that a small number of previous studies have examined the indirect effect of *bianzhi* on turnover intention among rural preschool teachers. Wang et al. (2022) found that *bianzhi* could moderate the effect of work pressure on the turnover intention of rural preschool teachers. Taking the above results together, we can conclude that *bianzhi* plays an important role in the formation of turnover intention among rural preschool teachers, especially for those in risky situations (e.g., facing negative workplace gossip and work stress).

### Practical implications

In addition to theoretical implications, our results also have practical implications. Based on the empirical findings, the managerial implications of the current study can be divided into three categories. First, our findings suggest that organizations should take practical measures to prevent and control negative workplace gossip to minimize its influence on the targets. Organizations could create a cultural atmosphere of zero tolerance toward negative workplace gossip by establishing guidelines or norms forbidding negative gossip (e.g., prohibiting talking about other teachers’ privacy). Accordingly, there should be penalties for those who break the guidelines and cause significant adverse effects within the organization, such as undermining team cohesion and destroying trust among colleagues. At the same time, given that negative workplace gossip is especially prevalent in an environment where information transparency is lacking (DiFonzo and Bordia, 2007), organizations should endeavor to build efficient communication channels for information exchange. For example, organizations could reveal accurate information regarding teachers’ interests promptly, reducing the likelihood of negative workplace gossip. Furthermore, managers could organize regular team communication and team-building activities to create constructive communication opportunities for teachers so as to promote mutual understanding, emotional connection, and team cohesion.

Second, our findings highlight the significance of preventing targets from experiencing ego depletion at work. Thus, organizations should take proper measures to save or replenish targets’ self-control resources. For example, organizations can provide teachers with self-affirmation training which may effectively promote teachers’ abilities of self-control and quick recovery from ego depletion (Yam et al., 2016). At the same time, organizations can help teachers replenish depleted resources by encouraging them to have short breaks at work. In addition, organizations can design employee assistance programs to help targeted teachers regulate negative emotions and cope with stress so as to prevent ego depletion caused by negative workplace gossip.

Third, our empirical findings also indicate that the local department of education can minimize the negative effects of negative workplace gossip and reduce the likelihood of turnover intention by increasing the supply of *bianzhi* for rural preschool teachers. Currently, preschool teachers have to compete with primary and secondary school teachers for *bianzhi* in most areas of China. In the future, the *bianzhi* of rural preschool teachers should be verified and allocated as a separate category to ensure the supply of *bianzhi* based on need. The department of education should also endeavor to raise the social status and improve the material welfare of rural preschool teachers without *bianzhi*, to buffer the effects of negative workplace gossip.

### Limitations and future research directions

It should be noted that there are some limitations in this study. Firstly, the cross-sectional study design makes it difficult to provide causal interpretations of the variables. Future research can adopt longitudinal designs or experimental studies to further test the moderated mediation model of negative workplace gossip affecting turnover intention. Secondly, convenience sampling was adopted in this study. This sampling method may lead to sampling bias, reducing the validity of the research. Random sampling should be used in future surveys. Thirdly, the present study used the self-report approach. This may cause social desirability effects, which probably impact the verifiability of the data and the authenticity of the results. Future research can address this problem by using multiple-source assessments. Lastly, in addition to ego depletion and *bianzhi*, there may be other susceptible factors involved in the effect of negative workplace gossip on turnover intention. Future research can continue to explore the mechanism of negative workplace gossip on the turnover intention of rural preschool teachers so as to provide practical references for suitable interventions for the prevention of teacher attrition in rural preschools.

## Conclusion

To sum up, our study found that negative workplace gossip was positively associated with rural preschool teachers’ turnover intention. Moreover, ego depletion played a mediating role in the relationship between negative workplace gossip and turnover intention and the mediating model was moderated by *bianzhi*. Specifically, the indirect effect was stronger for rural preschool teachers without *bianzhi* than for those with *bianzhi*. The findings can help to explain why and how negative workplace gossip is associated with turnover intention and enrich research on the mechanism of negative workplace gossip and turnover intention.

## Data availability statement

The raw data supporting the conclusions of this article will be made available by the authors, without undue reservation.

## Ethics statement

The studies involving human participants were reviewed and approved by Ethics Committee for Scientific Research of Hubei Normal University. Written informed consent to participate in this study was provided by the participants’ legal guardian/next of kin.

## Author contributions

CH proposed hypotheses, collected data, and completed paper writing. HW analyzed the data. All authors contributed to the article and approved the submitted version.

## Funding

This work was funded by Humanities and Social Sciences Research Project of the Ministry of Education of the People’s Republic of China (Grant No. 20YJC880025).

## Conflict of interest

The authors declare that the research was conducted in the absence of any commercial or financial relationships that could be construed as a potential conflict of interest.

## Publisher’s note

All claims expressed in this article are solely those of the authors and do not necessarily represent those of their affiliated organizations, or those of the publisher, the editors and the reviewers. Any product that may be evaluated in this article, or claim that may be made by its manufacturer, is not guaranteed or endorsed by the publisher.
